# Standardized Metadata for Human Pathogen/Vector Genomic Sequences

**DOI:** 10.1371/journal.pone.0099979

**Published:** 2014-06-17

**Authors:** Vivien G. Dugan, Scott J. Emrich, Gloria I. Giraldo-Calderón, Omar S. Harb, Ruchi M. Newman, Brett E. Pickett, Lynn M. Schriml, Timothy B. Stockwell, Christian J. Stoeckert, Dan E. Sullivan, Indresh Singh, Doyle V. Ward, Alison Yao, Jie Zheng, Tanya Barrett, Bruce Birren, Lauren Brinkac, Vincent M. Bruno, Elizabet Caler, Sinéad Chapman, Frank H. Collins, Christina A. Cuomo, Valentina Di Francesco, Scott Durkin, Mark Eppinger, Michael Feldgarden, Claire Fraser, W. Florian Fricke, Maria Giovanni, Matthew R. Henn, Erin Hine, Julie Dunning Hotopp, Ilene Karsch-Mizrachi, Jessica C. Kissinger, Eun Mi Lee, Punam Mathur, Emmanuel F. Mongodin, Cheryl I. Murphy, Garry Myers, Daniel E. Neafsey, Karen E. Nelson, William C. Nierman, Julia Puzak, David Rasko, David S. Roos, Lisa Sadzewicz, Joana C. Silva, Bruno Sobral, R. Burke Squires, Rick L. Stevens, Luke Tallon, Herve Tettelin, David Wentworth, Owen White, Rebecca Will, Jennifer Wortman, Yun Zhang, Richard H. Scheuermann

**Affiliations:** 1 J. Craig Venter Institute, Rockville, Maryland, and La Jolla, California, United States of America; 2 National Institute of Allergy and Infectious Diseases, Rockville, Maryland, United States of America; 3 University of Notre Dame, Notre Dame, Indiana, United States of America; 4 University of Pennsylvania, Philadelphia, Pennsylvania, United States of America; 5 Broad Institute, Cambridge, Massachusetts, United States of America; 6 Institute for Genome Sciences, University of Maryland School of Medicine, Baltimore, Maryland, United States of America; 7 Cyberinfrastructure Division, Virginia Bioinformatics Institute, Blacksburg, Virginia, United States of America; 8 National Center for Biotechnology Information, National Library of Medicine, Bethesda, Maryland, United States of America; 9 University of Georgia, Athens, Georgia, United States of America; 10 Kelly Government Solutions, Rockville, Maryland, United States of America; 11 Argonne National Laboratory, Lemont, Illinois, United States of America; 12 Department of Pathology, University of California San Diego, San Diego, California, United States of America; Emory University, United States of America

## Abstract

High throughput sequencing has accelerated the determination of genome sequences for thousands of human infectious disease pathogens and dozens of their vectors. The scale and scope of these data are enabling genotype-phenotype association studies to identify genetic determinants of pathogen virulence and drug/insecticide resistance, and phylogenetic studies to track the origin and spread of disease outbreaks. To maximize the utility of genomic sequences for these purposes, it is essential that metadata about the pathogen/vector isolate characteristics be collected and made available in organized, clear, and consistent formats. Here we report the development of the GSCID/BRC Project and Sample Application Standard, developed by representatives of the Genome Sequencing Centers for Infectious Diseases (GSCIDs), the Bioinformatics Resource Centers (BRCs) for Infectious Diseases, and the U.S. National Institute of Allergy and Infectious Diseases (NIAID), part of the National Institutes of Health (NIH), informed by interactions with numerous collaborating scientists. It includes mapping to terms from other data standards initiatives, including the Genomic Standards Consortium’s minimal information (MIxS) and NCBI’s BioSample/BioProjects checklists and the Ontology for Biomedical Investigations (OBI). The standard includes data fields about characteristics of the organism or environmental source of the specimen, spatial-temporal information about the specimen isolation event, phenotypic characteristics of the pathogen/vector isolated, and project leadership and support. By modeling metadata fields into an ontology-based semantic framework and reusing existing ontologies and minimum information checklists, the application standard can be extended to support additional project-specific data fields and integrated with other data represented with comparable standards. The use of this metadata standard by all ongoing and future GSCID sequencing projects will provide a consistent representation of these data in the BRC resources and other repositories that leverage these data, allowing investigators to identify relevant genomic sequences and perform comparative genomics analyses that are both statistically meaningful and biologically relevant.

## Introduction

Microbial and invertebrate vector genomes, indeed genomes in general, are being sequenced and deposited in public data repositories at an increasingly rapid pace [1], [2]. The scale and scope of pathogen sequencing projects are enabling the investigation of genotype-phenotype relationships, including the elucidation of genetic determinants of specific pathogen traits such as virulence and drug resistance [3]–[5]. Similarly, vector population genomic analyses are aiding in the development of novel control and prevention approaches and insecticide discovery [6], [7]. Rapid genome sequencing and analysis also allows the tracking of the origin and spread of new disease outbreaks in an unprecedented manner [8]. These genomics-based studies are only feasible if each sequence record is linked to meaningful metadata about the sequenced specimen. Unfortunately, inconsistencies in how the specimen source, clinical phenotypes, and sequence quality are described pose a significant barrier to these scientific inquiries. By standardizing metadata annotation and collection at the onset of a project, biologically meaningful epidemiologic, phylogenetic, and comparative genomic analyses can be performed [9]. Consistently applied metadata standards are also essential for retrospective study data integration and meta-analysis across studies. Future prospective studies can be designed to collect similar metadata fields to allow better integration with existing knowledge in the field. Thus, establishing metadata standards promotes maximal utility of the data generated and makes these data available for uses beyond what may have been originally envisioned.

Recognizing the need for better standardization of sequence-related metadata, the U.S. National Institute of Allergy and Infectious Diseases (NIAID) established a working group with representatives from NIAID, and the NIAID-funded Genomic Sequencing Centers (GSCIDs) (http://www.niaid.nih.gov/labsandresources/resources/dmid/gsc/Pages/default.aspx) and Bioinformatics Resource Centers (BRCs) (http://www.niaid.nih.gov/labsandresources/resources/dmid/brc/Pages/default.aspx) to develop an approach for capturing standardized genome sequence metadata. The GSCIDs work collaboratively with the research community to provide services for rapid and cost efficient production of high-quality genome assemblies and annotations, and high-throughput genotyping of NIAID Category A–C priority pathogens, microorganisms responsible for emerging and re-emerging infectious diseases, invertebrate vectors of infectious diseases, and related organisms. The BRCs manage, integrate, and display genome sequence data and annotation, as well as other research data types, including other “-omics” data (e.g. transcriptomics, proteomics, metabolomics), and data pertaining to epidemiology, surveillance, population genetics, genotype/phenotype association, antimicrobial resistance, and antigenicity for these pathogens and their vectors [10]–[15]. The BRCs also make available bioinformatics tools and services for processing, analyzing, and interpreting these data for further scientific investigation. The collaborative environment cultivated by these NIAID-supported projects presents a unique opportunity to ensure accurate and consistent metadata collection from sample providers, and rapid, transparent deposition of these data into publicly accessible resources, ensuring the availability of the data and required tools for effective mining and analysis of the sequence and associated metadata by the broader scientific research community.

Here we report on a multi-project and multi-institutional effort for the development of an approach for the capture of standardized human pathogen and vector sequencing metadata designed to support epidemiologic and genotype-phenotype association studies.

## Methods

In designing an approach for the capture of standardized metadata two important factors needed to be considered - what kind of information should be captured and how that information should be represented. These considerations can be largely addressed by specifying *(i) a minimum set of data fields* and *(ii) the controlled vocabularies or data dictionaries to be used as allowed values*. The data fields describe information about who performed the study, where the samples came from, when the samples were isolated, etc., for all sequencing projects, along the lines of the minimum information checklists established by the MIBBI Consortium [16]. These are ideally derived from established minimum information checklists, ensuring that the data is interoperable with data derived from other sequencing initiatives. The controlled vocabularies define the allowed values and acceptable formats for each data field. They are ideally derived from existing biomedical ontologies, ensuring that the same entities are described using the same terminologies that include embedded semantic relationships.

### Assembling Lists of Metadata Fields and Attributes

Beginning in May 2011, NIAID assembled a working group to develop an approach for capturing standardized genome sequence metadata – the GSCID-BRC Metadata Working Group. This working group consisted of representatives from the three GSCIDs – at the Broad Institute (http://www.broadinstitute.org/science/projects/gscid/genomic-sequencing-center-infectious-diseases), the J. Craig Venter Institute (http://gsc.jcvi.org), and the University of Maryland, School of Medicine, Institute for Genome Sciences (http://gscid.igs.umaryland.edu) - and the five Bioinformatics Resource Centers (BRCs) - the Eukaryotic Pathogen Database Resources (EuPathDB: http://EuPathDB.org), the Influenza Research Database (IRD: http://www.fludb.org/), the Pathosystems Resource Integration Center (PATRIC: http://patricbrc.org), the Bioinformatics Resource for Invertebrate Vectors of Human Pathogens (VectorBase: https://www.vectorbase.org), and the Virus Pathogen Resource (ViPR: http://www.viprbrc.org/). Importantly, the group focused on developing metadata standards that was congruous with other standards to avoid adding additional confusion to what has become a complicated landscape of biomedical data standards. The adopted approach consisted of developing an “application metadata standard”, which was derived through the collection of data fields and through mapping these fields wherever possible to synonymous terms existing in established “reference data standards” and biomedical ontologies. We therefore focused on developing a cross-compatible application standard to capture the relevant information describing a sequencing project, and then represent it in a standardized way. Such an approach could be used to guide the collection, representation, transmission, submission, and search of metadata relating to GSCID and BRC projects.

The process of developing the application standard began by establishing subgroups based on various areas of expertise (i.e. insect vector, eukaryotic pathogen, bacterial pathogen, and viral pathogen). Each subgroup then reviewed various internal and external sources of sequencing project and sample metadata to identify terms that were relevant at either the project or sample level. Names, descriptions, synonyms, allowed values, and other information were compiled for each metadata term and each was evaluated for its importance relating to data access and analysis use cases. Existing ontologies were then identified to further standardize the representation of the metadata fields. The separate lists of project-level and sample-level fields from all subgroups were then merged together and redundancy eliminated.

**Figure 1:**
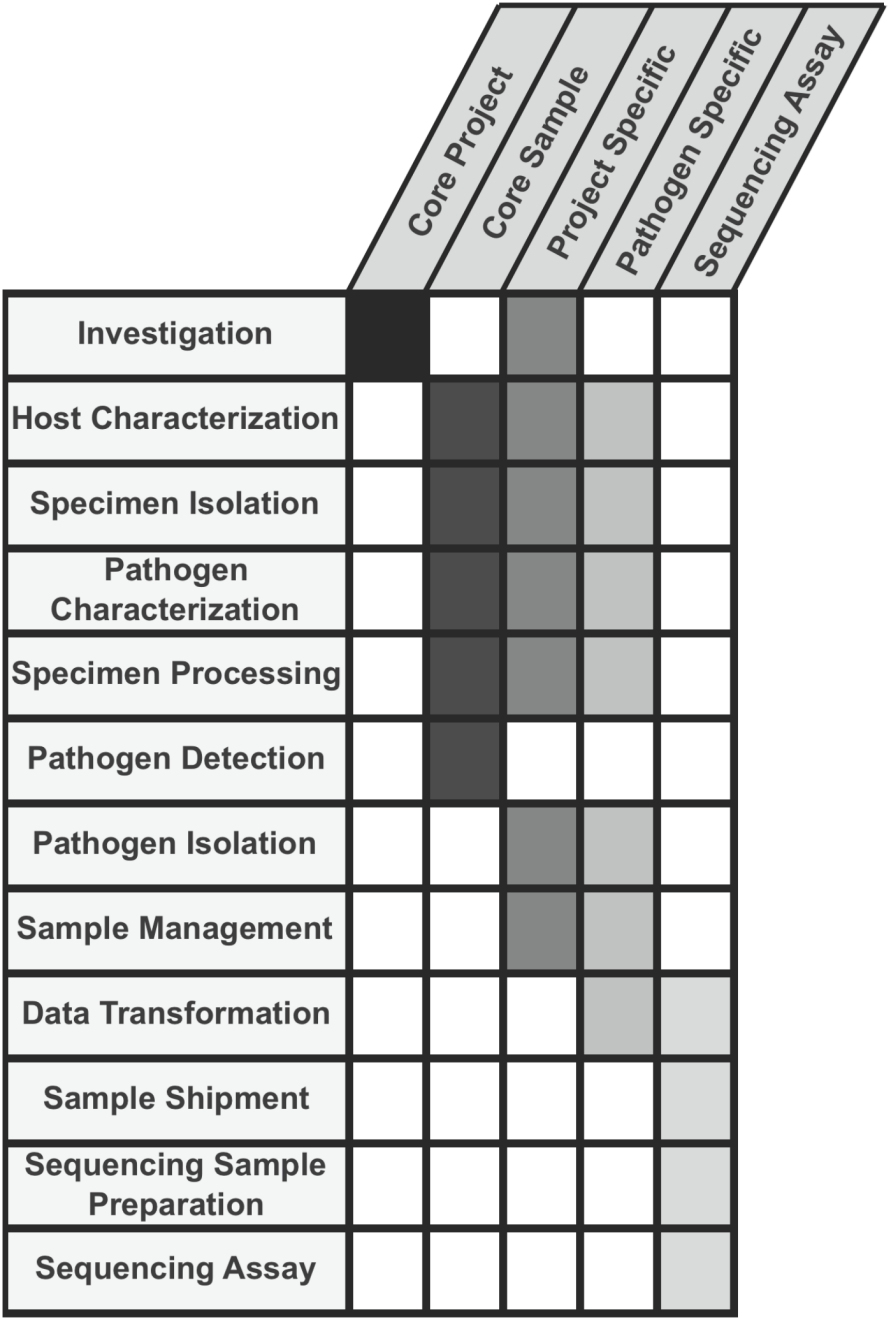
NIAID GSCID/BRC Project and Sample Application Standard Overview. Coverage of the twelve major data categories in the five data field collections is shown.

The final outcome of these efforts resulted in a set of metadata fields and associated descriptive information organized as being relevant to one of the following hierarchical groupings: *core project metadata* (metadata that applies to *all* projects), *core sample metadata* (metadata that applies to *all* samples), *sequencing assay metadata, pathogen-specific metadata,* and *project-specific metadata* (Figure 1). Submission of values for all core data fields would be required for all sequencing projects, with “not available” accepted as an allowed value for certain fields. Pathogen-specific and project-specific data fields would be made available as pick lists to provide additional optional information of relevance for a given project.

### Semantic Representation and Harmonization with Related Standards

As part of our effort to develop an application metadata standard, a comprehensive evaluation was undertaken to evaluate the degree of overlap between the draft collection of GSCID/BRC metadata fields derived above and those supported in other relevant data standards. As a result of this evaluation, the Ontology for Biomedical Investigations (OBI) [17] was adopted as the underlying framework because of its domain coverage, its adoption by other database resources, and the value of using its semantic formalism. OBI is the only member of OBO Foundry [18] collection of ontologies that covers all aspects of a biomedical investigation, and includes descriptions of the various protocols, processes and participants used in research. By incorporating the OBI ontology into the GSC/BRC metadata standard, the information represented would be directly comparable with other data represented using OBI and other OBO Foundry ontologies.

OBI is organized around processes used in biomedical investigations. To place the draft metadata fields into the OBI framework, we collaborated with OBI developers to first organize the metadata fields into the following planned processes: *investigation*, *specimen collection*, *sequencing assay*, and *data analysis*. Next, corresponding OBI (or other OBO Foundry ontology) terms were identified as either exact matches for a metadata field or as being an “is_a” parent in the OBI ontological hierarchy. If an equivalent term did not exist, a request was made to the OBI developers to create a new ontology term by providing an OBI-compatible label, a community preferred GSCID-BRC label as an exact synonym, a textual description and a logical definition (including parent class) to the OBI Issue Tracker for discussion and eventual adoption by OBI developers. This process was repeated until each draft GSCID-BRC data field was represented by an OBI (or other OBO Foundry) term. Thus, while the official labels, definitions, and unique identifiers of the data fields would be derived from the OBI ontology, the community preferred labels and descriptions of the draft metadata fields, which are both understandable and intuitive to the infectious disease community, would also be available. A graphical semantic representation was generated using the cmap tools (http://cmap.ihmc.us) to visualize the Core Project and Core Sample metadata fields as an ontological model. After reviewing the modeled representation, a web ontology language (OWL) file was generated as a GSCID/BRC View of OBI and posted at the NCBO BioPortal ontology repository website (http://bioportal.bioontology.org/ontologies/2127).

In addition, direct comparisons were made to identify synonymous terms from the Minimum Information about any (x) Sequence (MIxS) standard, which was developed by the Genomic Standards Consortium [19], as well as the NCBI BioProject and BioSample repositories [20] through an active collaboration with representatives from these two initiatives. The one-to-one mapping results between these existing standards and our application standard were included in the GSCID/BRC core metadata representation to facilitate cross-compatibility. MIxS comparisons were reviewed and discussed with the Genomic Standards Consortium to determine correctness. Feedback was also solicited from NCBI regarding the metadata field mapping to BioProject and BioSample as these would ultimately be used in the submission process to the respective NCBI repositories. This harmonization process identified additional metadata fields that covered aspects of study design and were consequently added to the core metadata representation prior to submission to the OBI Issue Tracker for inclusion in OBI.

In this way, all data fields identified as being relevant for pathogen/vector sequencing projects were represented with OBI or other relevant OBO Foundry ontology (e.g. Environment Ontology (EnvO)) terms, with mappings to equivalent terms in the MIxS standard where appropriate, and all data required for NCBI BioProject and BioSample registration were included in the resulting “GSCID/BRC Project and Sample Application Standard”. By providing this one-to-one mapping between these related standards, data represented using the GSCID/BRC Project and Sample Application Standard will be interoperable with related data represented using these other relevant standards.

### Evaluation and Refinement

Following the harmonization of metadata submission fields with existing initiatives, initial versions of Core Project, Core Sample, and Project-Specific metadata submission templates were established for use with all GSCID and BRC related projects. A metadata submission workflow was also defined to describe who would be responsible for providing a given data field and at what point the information would be provided in the sequencing and submission process. The GSCID/BRC Project and Sample Application Standard metadata submission templates (version 1.1) were then distributed to collaborating research scientists working on bacterial, viral, eukaryotic and vector sequencing projects for evaluation and feedback during a test submission. Their feedback was evaluated and incorporated into a revised version (1.2) of the Core Project and Core Sample data submission templates.

Upon completing the list of terms comprising version 1.2 of the GSCID-BRC standardized metadata collection, we again approached the personnel associated with the MIxS and NCBI data standards initiatives to encourage the adoption of missing terms while simultaneously ensuring the correctness of the mappings. This exercise helped us identify 15 terms that were directly related to a sequencing assay, and are either required fields associated with a Sequence Read Archive (SRA) submission or structured comments within a GenBank record. Consequently, these terms were segregated from the Core Sample section and consolidated into a new Sequencing Assay component to maintain its modular structure, resulting in Version 1.3 of the GSCID/BRC Project and Sample Application Standard. This workflow will ensure that the Core Project and Core Sample fields can be universally applicable regardless of the experimental assay used to produce the data, while simultaneously ensuring their usefulness in downstream analyses and maintaining compatibility with existing metadata standards. An archive of current versions of the GSCID/BRC Project and Sample Application Standard and all metadata submission templates are available from http://www.niaid.nih.gov/LabsAndResources/resources/dmid/Pages/metadatastandards.aspx.

## Results

### Resulting GSCID/BRC Project and Sample Application Standard

The resulting application standard comprises specific collections of standardized metadata fields divided into sections (Figure 1). Two sections are relevant to all pathogen/vector related research projects and sample collections and are thus considered “core”. One section includes data fields of relevance to the sequencing assay component of the workflow. Two sections are specific to a particular pathogen/vector category and are relevant to all projects related to that pathogens/vectors category (pathogen specific). One section includes fields that are project specific. A summary of each of these sections follows:

- The *Core Project* section includes 23 data fields that pertain to the overall project/investigation. This section includes items such as the project title, project rationale, contact information for investigators, links to any related publications, *etc*. (Table 1). Since all of these fields are relevant for any pathogen/vector-related project, they are considered to be “core” and would be required fields for a complete submission package. Ten of the 23 Core Project terms had equivalents in BioProject or MIxS.
- The *Core Sample* section contains 27 data fields grouped into five categories describing host characterization (e.g. species, age, sex and health status), specimen isolation (e.g. date and geographic location of specimen collection, specimen type and environmental source), pathogen detection (e.g. detected pathogen and method of detection), pathogen characterization (refers to the pathogenicity to humans), and specimen processing (samples from biosample repositories and sample identifier used by the source repository). Recognizing that investigators may not find the anticipated pathogen or may identify additional pathogens, the core sample section also captures details about *anticipated* species and their pathogenicity (Table 2). Since all of these fields are relevant for any pathogen/vector-related project, they are considered to be “core” and would be required fields for a complete submission package. The Core Sample terms had robust mapping to OBI, BioSample and MIxS, and most had equivalent OBO IDs.
- The *Sequencing Assay* section includes 10 data fields that are grouped into four categories describing sample shipment, sequencing sample preparation, sequencing assay and data transformation (post-sequencing steps, such as assembly and annotation) (Table S1 in File S1). Many of these fields would only be relevant for a subset of sequencing projects and so are considered optional.
- The *Pathogen Specific* section includes data fields that vary depending on the type of pathogen/vector: bacteria (Table S2 in File S1) or eukaryotic pathogen/vector (Table S3 in File S1). (Note that no data fields were identified that would be applicable for all virus sequencing projects given the large diversity of virus biology and genomic features, and so no virus-specific collection was assembled.) This section provides the possibility of including metadata specific to a particular pathogen/vector that do not necessarily apply to other types of pathogens/vectors, for example, extra chromosomal elements that may apply to some bacteria or eukaryotic pathogens, bacteria typing method, and malaria parasitemia measures.
- *Project Specific* data fields capture information not included in the previous sections but which investigators believe add relevant details about the investigation in certain circumstances. Since every project is different, these Project Specific fields are designed to capture those differences. To facilitate cross-study comparisons, a repository of Project Specific data fields is provided to enable interoperability through controlled vocabularies if and when a particular data field is relevant for a given project (Table S4 in File S1).

**Table 1:** Core Project Attributes.

FieldID	Field Name	Data Categories	OBO Foundry URL	BioProject Synonyms	MIxS Synonym
CP1	Project Title	Investigation	http://purl.obolibrary.org/obo/OBI_0001622	Title*	project name
CP2	Project ID	Investigation	http://purl.obolibrary.org/obo/OBI_0001628		
CP3	Project Description	Investigation	http://purl.obolibrary.org/obo/OBI_0001615	Description*	
CP4	Project Relevance	Investigation	http://purl.obolibrary.org/obo/OBI_0500000	Relevance*	
CP5	SampleScope	Investigation	http://purl.obolibrary.org/obo/OBI_0001884	Sample Scope*	
CP6	Target Material	Investigation	http://purl.obolibrary.org/obo/OBI_0001882	Material*	
CP7	Target Capture	Investigation	http://purl.obolibrary.org/obo/OBI_0001899	Capture*	
CP8	Project Method	Investigation	http://purl.obolibrary.org/obo/OBI_0001896	Methodology*	
CP9	Project Objectives	Investigation	http://purl.obolibrary.org/obo/OBI_0001892	Objective*	
CP10	Grant Agency	Investigation	http://purl.obolibrary.org/obo/OBI_0001942		
CP11	Supporting Grants/Contract ID	Investigation	http://purl.obolibrary.org/obo/OBI_0001629	Grant ID	
CP12	Publication Citation	Investigation	http://purl.obolibrary.org/obo/OBI_0001617	PubMed ID; DOI	ref_ biomaterial
CP13	Sample Provider PrincipalInvestigator (PI) Name	Investigation	http://purl.obolibrary.org/obo/OBI_0001889		
CP14	Sample ProviderPI’s Institution	Investigation	http://purl.obolibrary.org/obo/OBI_0001880		
CP15	Sample ProviderPI’s email	Investigation	http://purl.obolibrary.org/obo/OBI_0001903		
CP16	Sequencing Facility	Investigation	http://purl.obolibrary.org/obo/OBI_0001891		
CP17	Sequencing FacilityContact Name	Investigation	http://purl.obolibrary.org/obo/OBI_0001888		
CP18	Sequencing FacilityContact’s Institution	Investigation	http://purl.obolibrary.org/obo/OBI_0001897		
CP19	Sequencing FacilityContact’s email	Investigation	http://purl.obolibrary.org/obo/OBI_0001894		
CP20	Bioinformatics Resource Center	Investigation	http://purl.obolibrary.org/obo/OBI_0001626		
CP21	Bioinformatics Resource Center Contact Name	Investigation	http://purl.obolibrary.org/obo/OBI_0001883		
CP22	BioinformaticsResource CenterContact’s Institution	Investigation	http://purl.obolibrary.org/obo/OBI_0001881		
CP23	BioinformaticsResource CenterContact’s email	Investigation	http://purl.obolibrary.org/obo/OBI_0001887		

*Mandatory NCBI BioProject attributes.

**Table 2:** Core Sample Attributes.

FieldID	Field Name	Data Categories	OBO Foundry URL	BioSample Synonym	MIxS Synonym
CS1	SpecimenSource ID	Host Characterization	http://purl.obolibrary.org/obo/OBI_0001141	host_subject_id	host_ subject_id
CS2	SpecimenCategory	Pathogen Detection	http://purl.obolibrary.org/obo/OBI_0100051	sample_category	
CS3	Specimen SourceSpecies	Host Characterization	http://purl.obolibrary.org/obo/OBI_0100026	host*	host_taxid
CS4	Species SourceCommon Name	Host Characterization	http://purl.obolibrary.org/obo/OBI_0100026	Host_common_name	host_ common_ name
CS5	Specimen SourceGender	Host Characterization	http://purl.obolibrary.org/obo/PATO_0000047	host_sex	sex
CS6	Specimen SourceAge - Value	Host Characterization	http://purl.obolibrary.org/obo/OBI_0001167	host_age	age
CS7	Specimen SourceAge - Unit	Host Characterization	http://purl.obolibrary.org/obo/UO_0000003	host_age	
CS8	Specimen SourceHealth Status	Host Characterization	http://purl.obolibrary.org/obo/PATO_0001995	host_health_state	health_ disease stat
CS9	Specimen SourceDisease	Host Characterization	http://purl.obolibrary.org/obo/OGMS_0000031	host_disease*	disease status
CS10	Specimen CollectionDate	Specimen Isolation	http://purl.obolibrary.org/obo/OBI_0001619	collection_date*	collection date
CS11	Specimen CollectionLocation - Latitude	Specimen Isolation	http://purl.obolibrary.org/obo/OBI_0001620	lat_lon*	geographic location (latitude and longitude)
CS12	Specimen CollectionLocation - Longitude	Specimen Isolation	http://purl.obolibrary.org/obo/OBI_0001621	lat_lon*	geographic location (latitude and longitude)
CS13	Specimen CollectionLocation - Location	Specimen Isolation	http://purl.obolibrary.org/obo/GAZ_00000448	geo_loc_name*	
CS14	Specimen CollectionLocation - Country	Specimen Isolation	http://purl.obolibrary.org/obo/OBI_0001627	geo_loc_name*	geographic location (country and/or sea region)
CS15	SpecimenID	Specimen Isolation	http://purl.obolibrary.org/obo/OBI_0001616	sample_name*	
CS16	SpecimenType	Specimen Isolation	http://purl.obolibrary.org/obo/OBI_0001479	host_tissue_sampled	body habitat, body site, body product
CS17	SuspectedOrganism(s)in Specimen- Species	Pathogen Detection	http://purl.obolibrary.org/obo/OBI_0000925	organism*	
CS18	SuspectedOrganism(s)in Specimen- Subclassification	Pathogen Detection	http://purl.obolibrary.org/obo/OBI_0000925	strain*	subspecific genetic lineage
CS19	Human PathogenicityofSuspectedOrganism(s) in Specimen	Pathogen Characteristic	http://purl.obolibrary.org/obo/IDO_0000666	pathogenicity	phenotype
CS20	EnvironmentalMaterial	Specimen Isolation	http://purl.obolibrary.org/obo/ENVO_00010483	isolation_source*	environment (material)
CS21	Organism DetectionMethod	Pathogen Detection	http://purl.obolibrary.org/obo/OBI_0001624	organism_detection_method	sample collection device or method
CS22	SpecimenRepository	Specimen Processing	http://purl.obolibrary.org/obo/OBI_0001885	culture_collection	source material identifiers
CS23	Specimen RepositorySample ID	Specimen Processing	http://purl.obolibrary.org/obo/OBI_0001900	culture_collection	source material identifiers
CS24	Comments	Specimen Comments	http://purl.obolibrary.org/obo/OBI_0001898		
CS25	Specimen CollectorName	Specimen Isolation	http://purl.obolibrary.org/obo/OBI_0001895	collected_by*	
CS26	Specimen Collector’sInstitution	Specimen Isolation	http://purl.obolibrary.org/obo/OBI_0001893	specimen_collector’s_ institution	
CS27	Specimen Collector’semail	Specimen Isolation	http://purl.obolibrary.org/obo/OBI_0001890	specimen_collector’s_ institution	

*Mandatory NCBI BioSample attributes in the “Pathogen: clinical or host-associated” version 1.0 package.

### Semantic Representation

**Figure 2:**
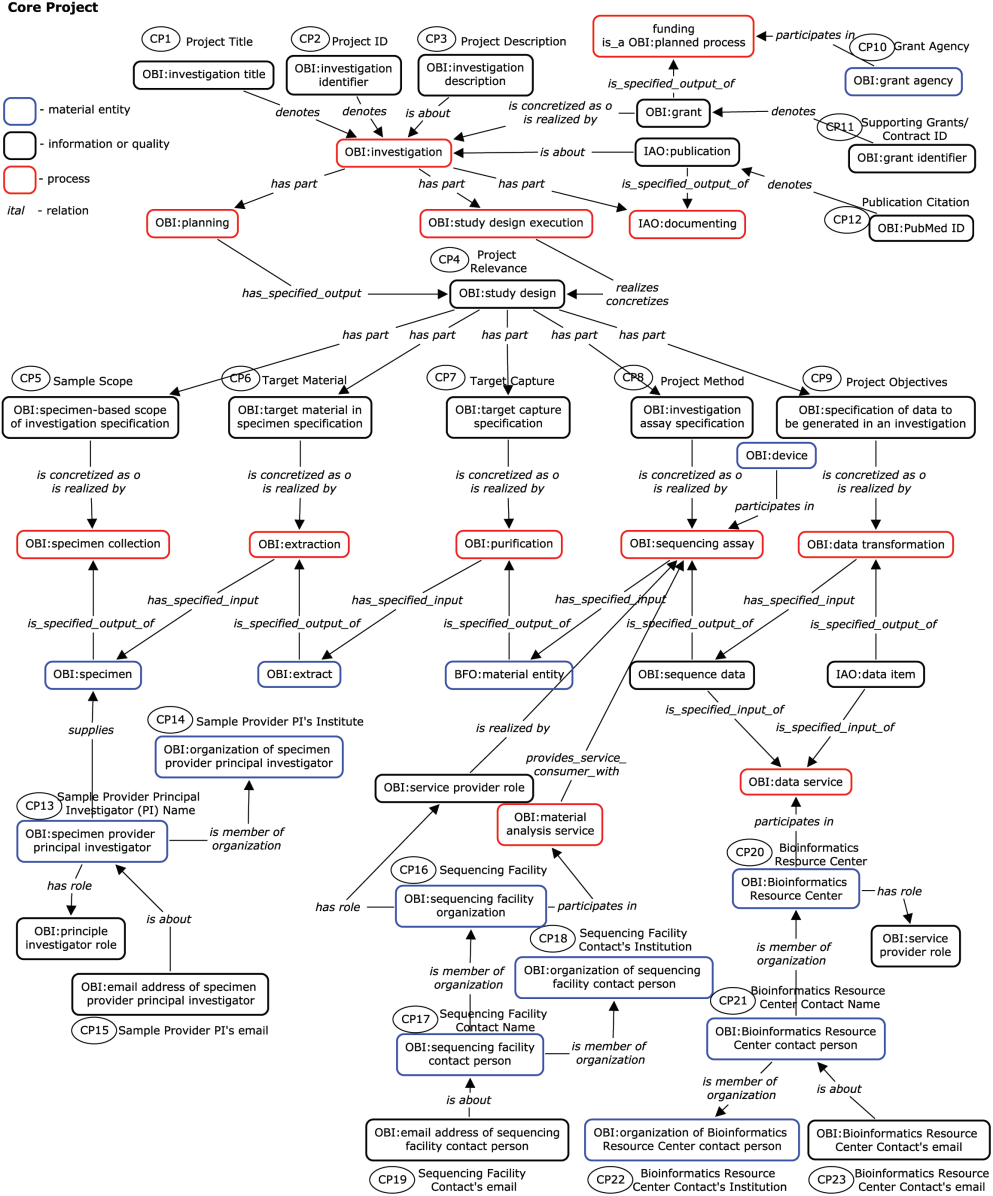
Semantic Network of the Core Project Data Fields. A semantic representation of the entities relevant to describe infectious disease projects based on the OBI and other OBO Foundry ontologies is shown. Distinctions are made between material entities (blue outlines), information entities and qualities (black outlines), and processes (red outlines). Entities are connected by standard semantic relations, in *italic*. The subset of entities selected as Core Project fields are noted with ovals containing the respective Field ID. For example, both the “Project Title” (CP1) and “Project ID” (CP2) *denote* an OBI:Investigation; the “Project Description” (CP3) *is_about* the same OBI:Investigation.

**Figure 3:**
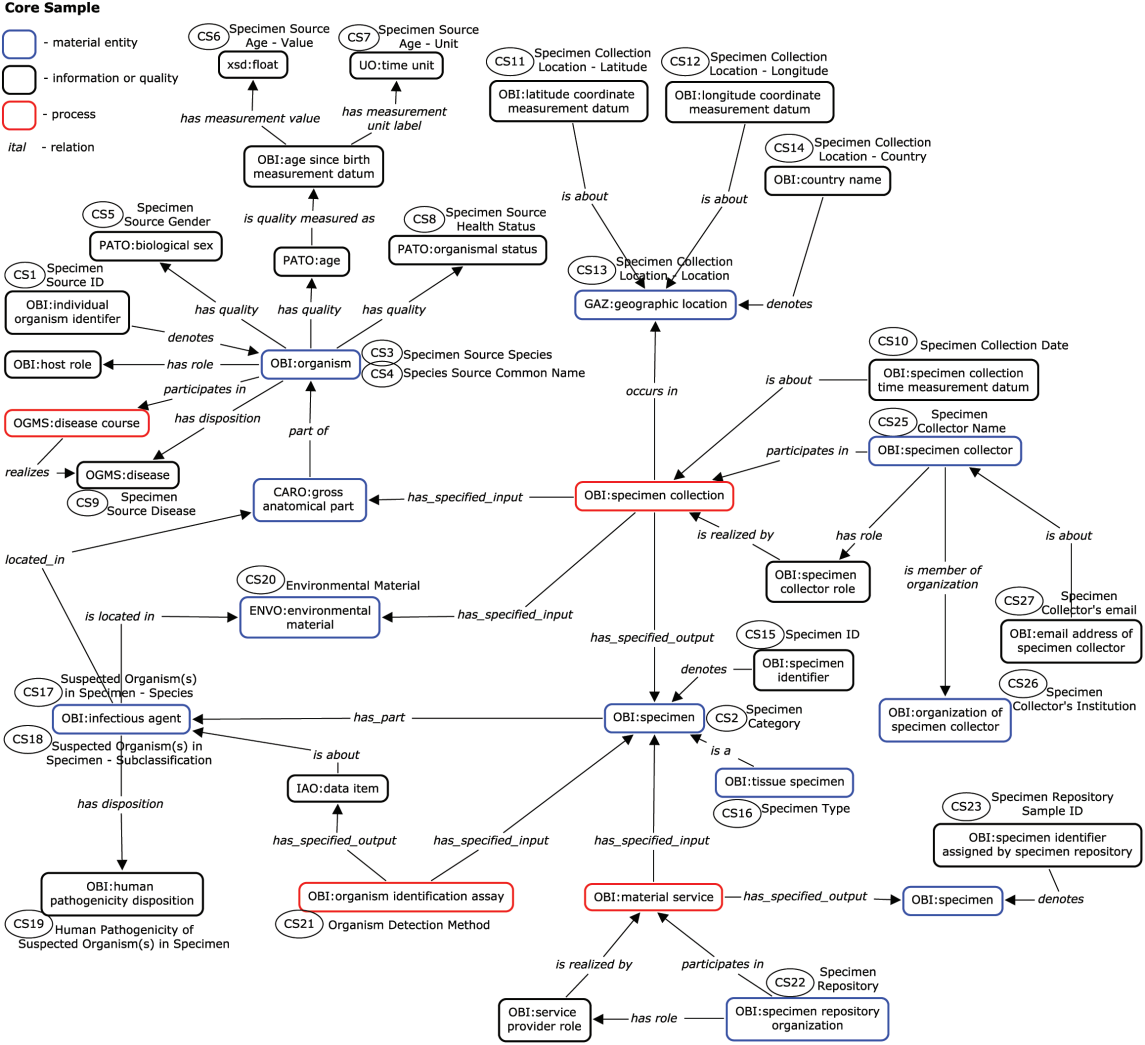
Semantic Network of the Core Sample Data Fields. A semantic representation of the entities relevant to describe infectious disease samples based on the OBI and other OBO Foundry ontologies is shown. Distinctions are made between material entities (blue outlines), information entities and qualities (black outlines), and processes (red outlines). Entities are connected by standard semantic relations, in *italic*. The subset of entities selected as Core Sample fields are noted with ovals containing the respective Field ID. For example, the OBI:organism *has_quality* “Specimen Source Gender” (CS5), which is equivalent to the PATO:biological sex, and *has_quality* PATO:age, and *has_quality* “Specimen Source Health Status” (CS8), which is equivalent to PATO:organismal status. PATO:age *is_quality_measured_as* OBI:age since birth measurement datum, which *has_measurement_value* “Specimen Source Age – Value” (CS6) and *has_measurement_unit_label* “Specimen Source Age – Unit” (CS7).

After the list of terms to be included in the Core Project, Core Sample, and Sequencing Assay data fields were compiled, they were then assembled into a semantic network based on OBI and other OBO Foundry compatible ontologies and relations (Figures 2 and 3). The goal of this process was to define the relationships between the various data fields and identify any gaps or inconsistencies that existed in the original list of data fields. For example, only one temporal data field for any given sequenced specimen was included in the first draft of Core Sample terms; however, it quickly became apparent that one time point was insufficient since a time measurement may be assigned when an infectious agent was first collected from the specimen source organism, when the health status of the specimen source was assessed, when the sample material was extracted from the specimen, when the sample material was subjected to an experimental assay, etc. (Figure 3). Indeed, all processes occur within their own timespan, and their temporal relationships can have important implications for the interpretation of the resulting sequence record. Although these temporal (and other) relationships are implied, constructing a formal semantic representation allowed us to correct similar omissions and clarify meanings that had previously been unintentionally ambiguous. In the case of Core Project, similar data fields were identified for each of the main parties involved – sample providers, assay centers and bioinformatics centers (Figure 2).

A second effect of this semantic network representation was that it generated a logical structure that reflects the processes being performed at various stages of scientific experimentation and data generation (e.g. specimen isolation, material processing, experimental assay and data processing), thereby delineating the data categories described above. Upon further examination, we found that the relationships existing within some of these sub-networks were not limited to sequencing experiments, thereby making the data structures for Core Project and Core Sample modular and reusable for other kinds of assays. Due to their general nature, the foundational structure of core metadata terms and their process categories could also be extended to define the relationships that exist between project-specific metadata terms.

## Discussion

Pathogen and vector genomic DNA and cDNA sequences have been deposited in open access sequence repositories since the creation of GenBank in the early 1980s [21]. The availability of genome sequence and related functional data has helped drive the further development of more specialized resources, like the BRCs, that facilitate the integration and analysis of these combined data [16]. The number of sequencing projects has dramatically increased due to the development of new sequencing technologies, the availability of computational resources, and the support for focused sequencing efforts, including those being conducted at NIAID-funded GSCIDs. The value of these large-scale genome projects, including the statistical robustness of downstream analyses, is influenced greatly by the quality of the associated metadata describing the characteristics of the sample used and the circumstances surrounding its collection.

The initiative described here focused on assembling an application standard to collect metadata for pathogen, parasite, and vector sequences. The working group has interacted with a broad collection of key stakeholders in the infectious disease research, genome sequencing, bioinformatics, and data standards communities to develop this application standard to ensure the continued relevance and usefulness of data standards. GSCID/BRC projects are now required to adhere to the metadata standard collections developed herein. Consequently, consistent metadata fields will accompany all viral, bacterial, and eukaryotic pathogen or vector samples currently being sequenced and annotated by the GSCIDs. All allowable metadata will be submitted to the appropriate public repository, with the remaining being made publicly available in the respective BRC. Current projects being undertaken at the GSCIDs involve diverse pathogens and vectors representing a wide range of geographic origins, temporal origins, and disease outcomes. Metadata collected in association with these efforts will be particularly important given the need to associate such population variation data with specific sample phenotypes that characterize ecology, behavior, physiology, genetic diversity, antigenic and allelic variation, as well as vector-pathogen interactions.

### Relationship to other Data Standards Initiatives

It is important to recognize that this application standard does not re-invent or recapitulate what is already available in other standards. Rather, our efforts focused on the harmonization and inter-compatibility with existing resources such as OBI, MIxS, and BioProject/BioSample [17], [20], [22], [23]. Our effort at harmonization is reflected in the fact that our Core Project and Core Sample collections include mappings to OBO foundry IDs and equivalent terms existing in OBI, BioSample/BioProject, and MIxS. This harmonization effort will allow data submitters to represent their sequencing metadata in a single schema that will be compatible with representations used by many other bioinformatics resources, including the BioSample/BioProject registrations required for GenBank submissions.

### Value of Modeling Semantic Representation

The representation of metadata using an ontology-driven semantic framework was a relatively novel feature of this approach. Assembling the data elements into a semantic framework provides interoperability between databases, minimizes the loss of information when transferring or converting between standards, and allows ontology-driven inference engines to take full advantage of this newly assimilated data as it is applied to other emerging “-omics” technologies. The semantic framework adopted for this purpose was largely based on the Ontology of Biomedical Investigation (OBI) [17], a newly-admitted OBO Foundry ontology focused on the representation of experimental planned processes and related entities [18]. OBI has been built on the high-level framework provided by the Basic Formal Ontology (BFO) that divides the universe into three main categories – occurrents (processes, with definable starts and ends), independent continuants (objects, that exist throughout time) and dependent continuants (characteristics of those objects) [24]. The semantic modeling described here enabled the visualization of the entire experimental workflow, from specimen collection to sequence submission, with terms of the component processes and participants involved.

### Tools for Standards-compliant Data Submission

In order to make it relatively easy for data providers to comply with the developed GSCID/BRC Project and Sample Application Standard, three different tools have been developed. First, each of the BRCs is making data submission spreadsheets available in Excel formats through their websites (e.g. http://www.fludb.org/brc/datastd and http://www.viprbrc.org/brc/datastd) since most potential data submitters are comfortable with using Excel spreadsheets for the capture of sample-level metadata. Second, an electronic data capture tool called O-Meta developed at the J. Craig Venter Institute has been configured to comply with the GSCID/BRC Project and Sample Application Standard and made freely available through GitHub at https://github.com/movence/ometa. Third, the PATRIC BRC is exploring the use of a Google spreadsheet widget called OntoMaton (https://github.com/ISA-tools/OntoMaton) that will enable the users to directly access relevant ontology terms using the ISA-Tab framework for metadata submission [25], [26]. This solution allows pre-defined ontology terms to be searched for and inserted in real-time via the NCBO BioPortal and the Ontology Lookup Service at the European Bioinformatics Institute [27]–[29]. These approaches should provide potential data submitters with user-friendly tools for both local metadata storage and metadata submission that are compliant with the developed data standard.

### Next Steps/Future Development

Although considerable discussion and revision went into the development of the released version 1.3 of the metadata standard, it was also recognized that ongoing refinement would likely be necessary to address future changes in the infectious disease field. Therefore, community members will be able to provide input on suggested enhancements to the submission templates through the GitHub repository at https://github.com/GSCID-BRC-Metadata-Standard-WG/GSCID-BRC-Project-and-Sample-Application-Standard. All requests will be periodically reviewed by the GSCID-BRC Metadata Working Group and updated versions of the templates released as appropriate.

Early in the process of developing the current application standard, we came to the realization that standardizing the representation of metadata relating to clinical encounters of human-derived samples would be relatively complex. A wide range of signs, symptoms, laboratory test results, and physical exam assessments could potentially be relevant while patient privacy and re-identification risk also have to be factored in. Since these issues would only be relevant for the subset of samples derived from human hosts, they do not fall into the category of core metadata fields. For these reasons, a separate working group was assembled to specifically deal with how to approach the standardization of clinical metadata. When completed, the standardized clinical metadata fields will be reported in a separate publication, and will be treated as project-specific fields for use on a case-by-case basis.

### Conclusions

The development of metadata standards for use by all GSCID sequencing projects will allow for a consistent representation of these data in the BRC resources and will also serve as a paradigm for other pathogen sequencing projects, thus supporting further interoperability. By capturing key information about pathogen isolates for the genome sequences being deposited in public data repositories in a consistent way, the standardized metadata will allow the pathogen research community to identify all representatives of genome sequences that match their particular interests, which will in turn allow them to perform statistically meaningful, and biologically relevant comparative genomics analysis. The end result will be a wealth of information about sequenced pathogens and vectors that can be used for more accurate downstream analysis.

## Supporting Information

File S1 **Supporting tables. Table S1,** Sequencing Assay Attributes. **Table S2,** Bacterial Pathogen-Specific Attributes. **Table S3,** Eukaryotic Pathogen- and Vector-Specific Attributes. **Table S4,** Project Specific Attributes.
(DOCX)
