# Supplementary material for: Standardized Metadata for Human Pathogen/Vector Genomic Sequences
Source: PLoS One. 2014 Jun 17;9(6):e99979. doi: 10.1371/journal.pone.0099979 (PMC4061050; doi:10.1371/journal.pone.0099979)
Supplement: File S1 — Supporting tables. Table S1, Sequencing Assay Attributes. Table S2, Bacterial Pathogen-Specific Attributes. Table S3, Eukaryotic Pathogen- and Vector-Specific Attributes. Table S4, Project Specific Attributes. (DOCX) [file pone.0099979.s001.docx]

**Supporting Information**

**Table S1. Sequencing Assay Attributes**

| **Sequencing Assay Field ID** | **Field Name** | **Data Categories** | **Description** | **OBO Foundry URL** | **MIxS Equivalent** |
| --- | --- | --- | --- | --- | --- |
| SA1 | Sample ID - Sequencing Facility | Sample Shipment | Unique identifier used by the relevant sequencing center to identify the sample submitted by the sample provider. | http://purl.obolibrary.org/obo/OBI_0001901 |  |
| SA2 | Nucleic Acid Extraction Method | Sequencing Sample Preparation | Experimental procedure used to derive the nucleic acid fraction from the submitted sample used for the sequencing reaction. | http://purl.obolibrary.org/obo/OBI_0666667 | sample material processing |
| SA3 | Nucleic Acid Preparation Method | Sequencing Sample Preparation | Details about the preparation of DNA samples for sequencing including if amplification was used (e.g., in the case of sequencing a single mosquito), and any other relevant molecular biology protocols done prior to sequencing. | http://purl.obolibrary.org/obo/OBI_0001902 | sample material processing |
| SA4 | Sequencing Technology | Sequencing Assay | Experimental procedure used to derive sequence data from the input assay sample including both method and device. Type of sequencing used based on approach (pyrosequencing) and technology (454). | http://purl.obolibrary.org/obo/OBI_0600047 | sequencing method |
| SA5 | Assembly Name | Data Transformation |  | http://purl.obolibrary.org/obo/OBI_0001948 |  |
| SA6 | Assembly Method | Data Transformation | Computational algorithm used to assemble individual sequence reads into larger contigs. Assembly details including but not limited to assembler type (overlap-layout-consensus, deBrujn), assembler version, any relevant QC information such as % known genes/ESTs captured. | http://purl.obolibrary.org/obo/OBI_0001522 | assembly |
| SA7 | Genome Coverage | Data Transformation | Depth of sequence coverage based both on external (e.g. Cot-based size estimates) and internal (average coverage in the assembly) measures of genome size. | http://purl.obolibrary.org/obo/OBI_0001618 | finishing strategy |
| SA8 | Annotation Provider | Data Transformation | The name of the responsible person, group or institution providing the set of annotated features for a genome sequences that is submitted to a resource such as GenBank. | http://purl.obolibrary.org/obo/OBI_0001947 |  |
| SA9 | Annotation Method | Data Transformation | The names and versions of the software and databases used in creating the set of annotated features that is submitted to a resource such as GenBank. | http://purl.obolibrary.org/obo/OBI_0001944 |  |
| SA10 | GenBank Record ID | Data Transformation | Unique identifier of the submitted GenBank sequence record(s). | http://purl.obolibrary.org/obo/OBI_0001614 |  |

**Table S2. Bacterial Pathogen-Specific Attributes**

| **Pathogen Specific Field ID** | **Field Name** | **Data Categories** | **Description** | **OBO Foundry URL** | **BioSample Synonym** | **MIxS Synonym** |
| --- | --- | --- | --- | --- | --- | --- |
| BAC1 | Bacteria Antibiotic Sensitivity | Pathogen Characteristic | Results of tests for antibiotic resistance, usually measured in minimum inhibitory concentration (MIC). Format: name of an antibiotic followed by 'MIC' other name of other metric and a measure of the quantity of antibiotic in ug/ml | http://purl.obolibrary.org/obo/IDO_0000470 |  | encoded traits |
| BAC2 | Bacteria Biovar | Pathogen Characteristic | Commonly used descriptor of distinguishing physical or biochemical characteristics of a bacterial population. |  |  | subspecific genetic lineage |
| BAC3 | Bacteria Chromosome Content | Pathogen Characteristic | Number of chromosomes in bacteria. | http://purl.obolibrary.org/obo/GO_0005694 |  | number of replicons |
| BAC4 | Bacteria Extra Chromosomal Elements | Pathogen Characteristic | Number of extrachromosomal elements in the organism. | http://purl.obolibrary.org/obo/OBI_0000430 |  | extra-chromosomal elements |
| BAC5 | Bacteria Pathovar | Pathogen Characteristic | Commonly used descriptor of distinguishing physical or biochemical characteristics of a bacteria. |  | pathovar | subspecific genetic lineage |
| BAC6 | Bacteria Serotype | Pathogen Characteristic | Serotype of the bacteria identified in the isolate sample. This is an identity determined by the data generated by the GSCID |  | serovar | subspecific genetic lineage |
| BAC7 | Bacteria Serotyping Method | Pathogen Characteristic | Experimental technique used to determine the serotype of the pathogen species in the isolate sample |  |  |  |

**Table S3. Eukaryotic Pathogen- and Vector-Specific Attributes**

| **Pathogen Specific Field ID** | **Field Name** | **Data Categories** | **OBO Foundry URL** | **BioSample Synonym** | **MIxS Synonym** |
| --- | --- | --- | --- | --- | --- |
| CE1 | Umbrella project ID(s) | Investigation | <http://purl.obolibrary.org/obo/OBI_0001628> |  |  |
| CE2 | Intended Sequence Repository(s) | Investigation |  |  | submitted_to_ insdc |
| CE3 | Submitter Name | Investigation | <http://purl.obolibrary.org/obo/OBI_0000068> |  |  |
| CE4 | subspecies/Subtype | Pathogen Characteristic |  |  |  |
| CE5 | Common name | Pathogen Characteristic |  |  |  |
| CE6 | Individuals (Number of males and females) |  |  |  |  |
| CE7 | Isolation, sampling or growth conditions (xenic/axenic culture,abcess aspirates,cysts) | Specimen Isolation |  |  |  |
| CE8 | Co-isolated organisms (in case this is a mixed culture) | Specimen Isolation |  |  |  |
| CE9 | Developmental Growth Stage (ie. sporozoite, male/female, or mixture of stages?) |  |  |  |  |
| CE10 | Date of sample collection for shipment to genomic sequencing center | Specimen Isolation |  |  |  |
| CE11 | Host Additional Classification - genotype | Host Classification | <http://purl.obolibrary.org/obo/OBI_0001305> |  |  |
| CE12 | Host Additional Classification - Strain | Host Classification |  |  |  |
| CE13 | Host Additional Classification - subtype | Host Classification |  |  |  |
| CE14 | Development stage |  |  |  |  |
| CE15 | Ploidy (ie. haploid, diploid, allopolyploid, polyploid or 1N, 2N, 3N etc...) | Pathogen Characteristic | [http://purl.obolibrary.org/obo/PATO_0001374](http://www.ontobee.org/browser/rdf.php?o=OBI&iri=http://purl.obolibrary.org/obo/PATO_0001374) |  | ploidy |
| CE16 | Number of replicons (chromosomes or segments) | Pathogen Characteristic |  |  | num_replicons |
| CE17 | Genome size estimate | Pathogen Characteristic |  |  | estimated_size |
| CE18 | Nucleic Acid Extraction Date | Sequencing Sample Preparation |  |  |  |
| CE19 | Extrachromosomal elements | Pathogen Characteristic |  |  | extrachrom_ elements |
| CE20 | Quantification (host/parasite; concentration and vol provided) |  |  |  |  |
| CE21 | Relevant Standard Operating Procedures (SOPs) | Data Transformation |  |  | sop |
| CE22 | Assembled genome size |  |  |  |  |
| CE23 | Relevant electronic resources |  |  |  | url |
| CE24 | Number of assembled contigs/scaffolds | Data Transformation |  |  |  |

**Table S4. Project Specific Attributes**

| **Project-Specific Field ID** | **Field Name** | **NCBI Component Name** | **NCBI Component Synonym** | **NCBI Component Definition** |
| --- | --- | --- | --- | --- |
| PS1 | Isolate | BioSample | isolate |  |
| PS2 | Host Disease Stage | BioSample | host_disease_ stage | Stage of disease at the time of sampling |
| PS3 | Host Disease Outcome | BioSample | host_disease_ outcome | Final outcome of disease, e.g., death, chronic disease, recovery |
| PS4 | Host Description | BioSample | host_description | Additional information not included in other defined vocabulary fields |
| PS5 | Specimen Voucher | BioSample | specimen_ voucher | Formal identifier of the Type Specimen of the source organism, usually stored in an institute collection. Name of the Institution and their internal Collection and/or sample codes. |
| PS6 | Genotype | BioSample | genotype | Observed genotype |
| PS7 | Serotype | BioSample | serotype | Taxonomy below subspecies; a variety (in bacteria, fungi or virus) usually based on its antigenic properties. Same as serovar and serogroup. e.g. serotype="H1N1" in Influenza A virus CY098518. |
| PS8 | Serovar | BioSample | serovar | Taxonomy below subspecies; a variety (in bacteria, fungi or virus) usually based on its antigenic properties. Same as serovar and serotype. Sometimes used as species identifier in bacteria with shaky taxonomy, e.g. Leptospira, serovar saopaolo S76607 (65357 in Entrez). |
| PS9 | Pathotype | BioSample | pathotype | Some bacterial specific pathotypes (example Escherichia coli - STEC, UPEC) |
| PS10 | Passage History | BioSample | passage_history | Number of passages and passage method |
| PS11 | Lab Host | BioSample | lab_host | Host on which a parasite is maintained in the lab, which may not be the same as the natural host (example hamster cells used to support a parasite normally found in mouse in the wild). Also used to list the bacterial strain in which a plasmid construct library is maintained in the lab (e.g. ID:167152 lab-host DH10B T1-resistant). |
| PS12 | Subgroup | BioSample | subgroup | Taxonomy below subspecies; sometimes used in viruses to denote subgroups taken from a single isolate. |
| PS13 | Subtype | BioSample | subtype | Used as classifier in viruses (e.g. HIV type 1, Group M, Subtype A). |
